# Brain Signatures of New (Pseudo-) Words: Visual Repetition in Associative and Non-associative Contexts

**DOI:** 10.3389/fnhum.2018.00354

**Published:** 2018-09-04

**Authors:** Beatriz Bermúdez-Margaretto, David Beltrán, Fernando Cuetos, Alberto Domínguez

**Affiliations:** ^1^Institute for Cognitive Neuroscience – Centre for Cognition & Decision Making, National Research University – Higher School of Economics, Moscow, Russia; ^2^Facultad de Psicología, Universidad de Oviedo, Oviedo, Spain; ^3^Instituto Universitario de Neurociencia (IUNE) and Facultad de Psicología, Universidad de La Laguna, Tenerife, Spain

**Keywords:** cluster-based permutation analysis, LPC, N400, reading, regression-based ERPs

## Abstract

The contribution of two different training contexts to online, gradual lexical acquisition was investigated by event-related potentials (ERPs) elicited by new, word-like stimuli. Pseudowords were repeatedly preceded by a picture representing a well-known object (semantic-associative training context) or by a hash mark (non-associative training context). The two training styles revealed differential effects of repetition in both behavioral and ERPs data. Repetition of pseudowords not associated with any stimulus gradually enhanced the late positive component (LPC) as well as speeded lexical categorization of these stimuli, suggesting the formation of episodic memory traces. However, repetition under the semantic-associative context caused higher reduction in N400 component and categorization latencies. This result suggests the facilitation in the lexico-semantic processing of pseudowords as a consequence of their progressive associations to picture-concepts, going beyond the visual memory trace that is generated under the non-associative context.

## Introduction

Visual recognition of familiar words is characterized by high accuracy and speed, as letter identification is achieved by means of direct, parallel processing. In contrast, reading unfamiliar words entails more cognitive effort and is error-prone, as a serial, sublexical decoding is required (Weekes, 1997; Ellis et al., 2009). It is generally accepted that to pass from a sublexical reading to the use of a direct visual recognition, a repeated visual exposure to words is necessary (Share, 1995, 1999; Dumay and Gaskell, 2007; Maloney et al., 2009). By means of repetition, a representation of the lexical item is formed in the reader’s orthographic lexicon; this becomes essential for reading it more efficiently in later encounters. Importantly, the lexical quality of these orthographic representations depends not only on the repeated visual exposure but also on other factors, such as their association with meaning (Perfetti and Hart, 2002). In this sense, although the formation of the word form through visual repetitions is indispensable for achievement of reading fluency, an association with a meaningful stimulus is also required (Coltheart et al., 2001). Therefore, training new words by combining both visual repetition and meaning association could contribute to their better integration in the reader’s cognitive system, leading to a more interactive processing and direct visual recognition.

Nevertheless, different stances can be found in the literature about the dependency on semantics in the lexicalization process of trained stimuli. On the one hand, some behavioral studies suggest that lexical representations might be formed after simple visual training in a relatively fast process. For instance, reductions in the length effect have been described after a few repetitions of new word-like stimuli – pseudowords – a process that has been interpreted as reflecting reading automatization due to pseudoword incorporation into the reader’s lexicon (Maloney et al., 2009; Kwok and Ellis, 2015; Kwok et al., 2016; Suárez-Coalla et al., 2016). In this sense, visual repetition is thought to be enough to promote the mental representation of new words and consequently its direct visual recognition, instead of the serial processing that is generally modulated by stimulus length. Furthermore, it has been found that visual repetition causes the interference in the processing of familiar words, in terms of higher categorization responses, when these stimuli were orthographic neighbors of the new words previously trained (Bowers et al., 2005; Wang et al., 2017). This interference effect is considered to reflect the establishment of the new stimulus into the reader’s lexicon, whose activation competes with the representation of the familiar word during its categorization, with the consequent increment in response times. On the other hand, different behavioral studies have claimed that visual repetition is not enough to reach fully lexicalized representations of new words. In these studies, data suggest that the nature of the orthographic representations formed after visual repetition could be episodic rather than lexical (Breitenstein et al., 2007; Clay et al., 2007; Qiao and Forster, 2013; Qiao et al., 2013; Tamura et al., 2017). This argument is based on the absence of lexical competition effects after simple visual repetition. More specifically, the reduction of the priming effect that would be expected when familiar words are primed by trained stimuli and both are orthographic neighbors (known as reduction of the prime-lexicality effect) is not found after visual repetition but only after the combination of visual and semantic training. Consequently, it is proposed that a multimodal training is required for the complete lexicalization of these stimuli, which ensures the achievement of a similar processing level – and hence interaction – between trained and already lexicalized words (Leach and Samuel, 2007).

Therefore, behavioral studies show some controversy regarding the episodic or lexical nature of the orthographic traces formed after repeated visual training and the need for semantic association for the lexicalization of new stimuli. However, it must be taken into account that behavioral approaches cannot fully address the question about which is the exact nature – episodic or lexico-semantic – of the processing achieved under different training procedures. In contrast, more fine-grain techniques, such as the recording of event-related brain potentials (ERPs), have demonstrated to be more appropriate to explore the mechanisms underlying the formation of new mental representations under visual and semantic trainings. Ultimately, this approach could clarify which is the most advantageous procedure for the lexicalization of new words.

In this line, ERPs research supports the distinction between episodic traces formed after visual training and lexico-semantic representations reached after semantic training. In particular, two ERP components have been found to be affected by these two types of stimulus trainings. First, repeated visual exposure produces changes in the activity of the late positive component (LPC), regardless of the type of stimulus: words (Van Strien et al., 2005), pseudowords (Swick and Knight, 1996), visual patterns (Van Petten et al., 2000), or pictures (Friedman, 1990). This component is characterized as a middle-posterior distributed potential that peaks around 500–700 ms post-stimulus onset and associated with episodic memory processes and recognition of previously presented stimuli (Rugg and Curran, 2007). In a previous study (Bermúdez-Margaretto et al., 2015), we found that LPC amplitudes positively covaried with pseudoword repetition, and that at the end of the training, both pseudowords and familiar words showed similar LPC amplitudes. This study showed that repeated visual training promotes episodic memory traces for pseudowords, allowing for their better recognition and categorization. However, it was concluded that visual repetition is likely not enough to integrate the new traces into the linguistic system of readers, as no effects reflecting the modulation of lexical processes were found after this training, supporting some previous claims from behavioral data.

Nonetheless, most ERP studies have focused on semantic associative training, reporting modulations of the N400 component. Specifically, these studies provide a semantic training for pseudowords by associating them with pictures (Dobel et al., 2009; Angwin et al., 2014) or definitions (Perfetti et al., 2005; Bakker et al., 2015), or embedding them in meaningful sentence contexts (Mestres-Missé et al., 2007; Borovsky et al., 2010; Frishkoff et al., 2010; Batterink and Neville, 2011). The main finding is that the previous presentation of meaningful stimulus might transfer semantic content to the pseudoword, leading to reductions in the N400 amplitude. This component has traditionally been related to the semantic processing of the stimuli and is sensitive to factors such as lexical status or frequency of words (Kutas and Hillyard, 1984; Bentin, 1987; Van Petten, 1993). Consequently, N400 amplitude reductions for meaningful trained pseudowords are considered to index their association with meaning and possibly their integration into the reader’s lexicon.

Therefore, prior electrophysiological research enables to highlight two critical statements about the processing achieved for new words under both trainings. On the one hand, visual repetition produces enhanced mental traces for pseudowords, which promote their efficient reading and categorization; however, the nature of these traces seems to be episodic rather than lexical, taking into account the LPC enhancements across this training. On the other hand, the association to a meaningful cue might be a requirement for the integration of these stimuli into the linguistic system and hence for their complete lexicalization, as is indicated by the reduction of N400 activity after combined training. However, none of these ERP studies have established a direct comparison between the effects produced by the semantic association of pseudowords and those found after their simple visual repeated exposure. Instead, they compared semantic training to no training (Perfetti et al., 2005; Dobel et al., 2009) or to training under semantically inconsistent contexts (Mestres-Missé et al., 2007; Borovsky et al., 2010; Frishkoff et al., 2010; Batterink and Neville, 2011). Therefore, no comparison has been reported between the brain modulations produced after the visual and the semantic training of new words, clarifying the advantage of one training over the other in the lexicalization of these stimuli. Furthermore, this prior research explores the effects in post-training tasks; thus, no information is offered about online changes produced in brain activity as a consequence of each training.

In our study, we aimed to compare the brain activity elicited by repeated exposure of new (pseudo-) words under two different contexts: a semantic-associative one, in which these stimuli were preceded by a picture of a known object and a non-associative one, in which pseudowords were preceded by a hash mark. These marks were used to introduce a control for trials procedure, enabling the maintenance of a similar presentation pattern between both conditions and ultimately comparison of the effect of the semantic-associative repetition with a simple repetition. We hypothesized that repetition of pseudowords under the semantic-associative context could improve not only visual but also lexico-semantic processing of pseudowords and enhance categorization responses across the task, modulating the amplitude of the N400 component. In contrast, simple repetition of pseudowords under the non-associative context would produce an improvement in the episodic rather than the lexico-semantic processing of these stimuli and, therefore, cause modulation of the LPC component. To test these hypotheses, we used a multiple linear regression analysis of the electroencephalographic (EEG) data to estimate ERP waveforms, a method that allows inclusion of continuous variables in the design, such as the number of repetitions in the current study, and considers individual variability in the data (Hauk et al., 2006, 2009; Smith and Kutas, 2015). Importantly, unlike classical ERPs computation methods, this new approach allows us to disentangle simultaneous modulations on ERP data by extracting independent estimates of relevant effects – in this case, those produced by repeated exposure to pseudowords under the two different training contexts.

## Materials and Methods

### Participants

Twenty-two students from the University of Oviedo, Spain (18 females; mean age of 22.09 years) took part in the experiment for course credits. All were native Spanish speakers, had normal or corrected-to-normal vision, and were right-handed according to the Edinburg Handedness Inventory (Oldfield, 1971). No neurological or psychiatric disorders were reported by participants. This research was approved by the Ethics Committee of the Psychology Department of the University of Oviedo. Before starting the experimental task, participants received pertinent information about the purpose of the study, the task, and their duration. Then, written informed consent was received from participants.

### Materials

Four hundred and forty-eight stimuli were included in a lexical decision task divided into six blocks. Sixty-four were experimental pseudowords, presented repeatedly across task blocks (once in each block). The remaining 384 stimuli were filler words, each presented one time only in sets of 64 through the task blocks. These stimuli served exclusively to construct the task, and were neither repeated nor included in the analyses.

In addition, half the words and pseudowords presented at each block were associated with a meaning by means of a preceding picture, whereas the other half was preceded by a hash mark (**Figure 2**). Therefore, each task block included 128 stimuli of four different types: words and pseudowords under a semantic-associative condition (which enabled the establishment of an association between the previous picture and the subsequent stimulus) and words and pseudowords under a non-associative condition (where the previous mark did not enable the association or prediction of the subsequent stimulus). More specifically, the same pseudowords were consistently preceded by the same cues – pictures or hash marks – whereas filler words were associated with those pictures that corresponded with their meaning. This enabled preservation of coherence between concepts represented by words and pictures. Thus, different pictures were presented in each block, in association with their corresponding word. Pictures were selected from the Snodgrass and Vanderwart (1980) set, and both pictures and hash marks had similar dimensions (15 × 10 cm). In addition, pseudowords under both semantic-associative and non-associative conditions were matched on different psycholinguistic variables by means of the BuscaPalabras database (Davis and Perea, 2005), to avoid the influence of sublexical effects on the earliest ERP components (**Table 1**).

**Table 1:** Matching means of each variable for pseudowords repeated under both semantic-associative and non-associative conditions (standard deviations are shown in brackets).

	Bigram frequency	First syllable frequency	Orthographic neighbors	Length of letters	Length of syllables
Pseudowords under semantic-associative context	516.28 (262.79)	271.44 (274.02)	2.68 (3.71)	5.12 (0.75)	2.18 (0.39)
Pseudowords under non-associative context	515.32 (225.53)	306.66 (224.30)	1.31 (2.05)	5.56 (0.61)	2.43 (0.50)

### Procedure

Participants had to decide if the stimulus was a real (pressing the key ‘YES’) or a non-real word (pressing the key ‘NO’). The correspondence between keys and hands (right-left) was counterbalanced across participants. Before starting the experiment, participants performed eight training trials (two trials per type of stimuli).

Stimuli were displayed in black, 18-point, Courier New letters (words and pseudowords) or in black lines (pictures and hash marks) over a white background at the center of the computer screen, by means of the experimental software E-Prime 2.0 (Schneider et al., 2002). **Figure 2** shows the sequence of the stimuli presentation under both training conditions. All trials were presented randomly within each task block. Behavioural and electrophysiological data were recorded from each participant during the task.

### EEG Recording and Pre-processing

An actiCAP with 62 Ag/AgCl active electrodes (Brain Products GmbH, Gilching, Germany) was used to record the electroencephalogram. Two other active electrodes were placed on the mastoid bones to calculate an offline reference (signal was online referenced to Cz). The impedance of active electrodes was kept below 25 kΩ. Two conventional electrodes were placed on the supraorbital and infraorbital canthus of the left eye. Both EEG and EOG signals were amplified and digitized by an actiCHamp amplifier system at a 1000-Hz sampling rate. High and low pass filters at 0.1 and 100 Hz, respectively, and a notch filter at 50 Hz, were applied. An additional low pass band filter at 30 Hz was applied off-line before computing regression-based ERPs (rERPs).

EEG data preprocessing and analysis was conducted using custom scripts in MATLAB (Mathworks, Natick, MA, United States), drawing on Fieldtrip Toolbox (Oostenveld et al., 2011) functions for artifact correction (exclusion criteria at ±100 μV), independent component analysis, cluster-based permutation statistics, and plotting waveforms and topographical representations. The EEG signal was segmented from 600 ms before to 1000 ms after the target onset, and the 250 ms preceding the picture/hash mark onset was used as baseline. These segments served as input for the single-trial regression framework used to analyze EEG signal (see section “Analysis of regression-based ERPs”). They also were averaged to obtain “classical” ERP waveforms of each experimental condition, which were used to construct graphics (**Figure 2**) and to run complementary analyses on traditional ERP components.

### Analysis of Behavioral Data

Mean reaction times (RTs) from each subject were submitted to repeated measures ANOVAs with the type of training (semantic-associative and non-associative) and the repetition (first vs. sixth block) as within-subject factors. Only trials with correct responses and with latencies not exceeding ±2 standard deviations were included in these analyses (93.18% of the overall trials were analyzed in the behavioral data).

### Analysis of Regression-Based ERPs

EEG data segments from −600 to 1000 ms post-target onset were analyzed following a rERPs framework. In this approach, the activity at every data point (latency and electrode) is regressed for each subject to relevant predictors on a single-trial basis (Smith and Kutas, 2015). Thus, the effects of type of training and repetition, as well as of the interaction between them, were estimated using the following multiple regression model:

$$y_{\text{i}}=\beta_{1}x_{1\text{i}}+\beta_{2}x_{2\text{i}}+\beta_{3}x_{\text{3i}}+\beta_{4}x_{4i}+{\text{noise}}_{\text{i}},$$

where *y*_i_ stands for the amplitude value in trial *i*, *x*_i_ for the code of the predictors in the same trial, and β for the values (or beta-coefficients) to estimate. A treatment-coding strategy was followed to define the different predictors within this model: predictor *x*1 (intercept) was always coded as 1, thereby serving as reference for other predictors, which in turn represented different treatment options or effects; predictor *x*2 (coded as 0 or 1), which is the difference between semantic-associative and non-associative trials regardless of the block number (global type of training effect); predictor *x*3 (coded in five steps of 0.2 each from 0 to 1), which is the repetition effect for the reference condition (e.g., semantic-associative trials); and predictor *x*4 (resulting from multiplying *x*2 and *x*3), which is the difference between repetition effects for semantic-associative and non-associative trials (interaction effect). Note that to correctly decompose the significant interaction effect, we needed to estimate rERPs corresponding to the repetition effect for both semantically associated and non-associated stimuli. This means that the regression model had to be computed twice – once with semantically associated pseudowords as reference and another with isolated pseudowords non-associated to any stimulus. This additional regression estimation allowed us to clarify the interaction by conducting additional contrasts on the identified interactive cluster(s). The regression model was applied for correct pseudowords, involving on average a total of 144 trials (74.50%) for pseudowords under the semantic-associative condition and 139 (72.44%) for pseudowords under the non-associative condition.

The resulting rERPs (or beta-coefficients) for the interaction effect were submitted to cluster-based random permutation analysis (Maris and Oostenveld, 2007). This non-parametrical approach allowed us to identify, over the whole rERP segment (here, 441 time points × 60 channels = 24,660 sample points), clusters (data points in close temporal and spatial proximity) in which the activity of these effect-related rERPs differed significantly from zero while effectively controlling for multiple comparisons (error type I). In the current study, this permutation approach was implemented as follows:

First, the beta-coefficients for the interaction effect were contrasted against zero by dependent sample *t*-tests computed for every sample point. Then, clusters were formed on the basis of spatial and temporal adjacency, selecting those data points which were below or equal to α level of 0.05 (here, a minimum of three adjacent sample points were required). Next, a cluster-level statistic was calculated for each observed cluster by taking the sum of all the individual *t*-values within it. A null distribution of the cluster-level statistic was calculated by randomly assigning rERPs segments (here, 1000 times) to the experimental condition. After each randomization, a new cluster-level statistic was calculated, and the one with the largest effect size was entered into the null distribution. The observed cluster was considered significant if the probability of the null hypothesis (namely, the proportion of cases in which values of this distribution are larger than the observed cluster-level statistic) was less than or equal to 5%.

Once a significant interactive cluster was detected with the above approach, its time interval and representative electrodes were used to average separately the rERP representing the repetition effects for pseudowords presented under both semantic-associative and non-associative contexts. To determine the significance and direction of the repetition effects, two contrasts compared the respective repetition effects (for semantically associated and isolated pseudowords) against zero. A third paired sample *t-*test contrasted both repetition effects to confirm the reliability and direction of their difference.

## Results

### Behavioral Data

**FIGURE 1:**
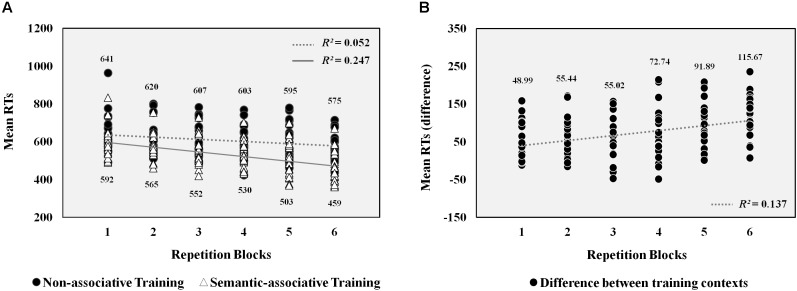
Left half **A**. Scatterplots of RTs obtained for pseudowords. Each marker indicates the mean RT obtained by each participant for pseudowords presented at both training contexts through the six repetition blocks. Numbers at the top and bottom of the scatterplots show the mean RTs obtained at each repetition block for pseudowords presented at the semantic-associative and non-associative conditions, respectively. *R*-squared coefficients indicate the proportion of the decrease in pseudowords’ mean RTs that is explained by repetitions across both training contexts. Dotted and solid lines show the linear trend followed by RTs under each condition. Right half **B**. Scatterplots of the differences between RTs obtained for pseudowords at both conditions. Each marker represents the difference between the mean RT obtained for pseudowords at both contexts by each participant across the six repetition blocks. Numbers at the top show the difference in mean RTs obtained between conditions at each repetition block. *R*-squared coefficient indicates the proportion of the increase in differences between the mean RTs obtained for pseudowords at both conditions that is explained by its repetition across blocks. Dotted line shows the linear trend followed by these differences.

**Figure 1A** shows mean RTs for pseudowords presented at both training contexts through each repetition block. The percentage of correctly responded pseudowords obtained per condition was above 90% (on average, from the first to the sixth repetition block, semantically associated pseudowords: 98.91, 98.50, 97.91, 97.63, 98.11, and 98.14%; non-associated pseudowords: 97.40, 96.81, 95.22, 96.97, 94.96, and 96.67%). Analysis carried out on latencies showed a significant interaction between type of training and repetition effects in analyses both by participants [*F*1(1,21) = 25.1, *p* < 0.001, η^2^ = 0.54] and by items [*F*2(1,127) = 79.9, *p* < 0.001, η^2^ = 0.39].

Follow-up analyses showed that pseudoword repetition under the non-associative condition caused a significant reduction in RTs across task blocks [*F*1(1,21) = 15.87, *p* < 0.01, η^2^ = 0.43; *F*2(1,127) = 66.71, *p* < 0.001, η^2^ = 0.51]. However, this reduction was greater for pseudowords associated with a picture [*F*1(1,21) = 49.78, *p* < 0.001, η^2^ = 0.7; *F*2(1,127) = 562.56, *p* < 0.001, η^2^ = 0.9]. As a consequence, significant differences between both types of pseudowords increased from the first block [*F*1(1,21) = 23.21, *p* < 0. 001, η^2^ = 0.52; *F*2(1,127) = 37.36, *p* < 0.001, η^2^ = 0.37] to the sixth [*F*1(1,21) = 102.5, *p* < 0.001, η^2^ = 0.83; *F*2(1,127) = 325.86, *p* < 0.001, η^2^ = 0.84]. Therefore, pseudoword repetition under the semantic-associative context produced a greater reduction in response times across blocks than under the non-associative context.

Additionally, regression analyses were conducted to explore further the causal relation between the repetition of pseudowords across blocks and the decrease in RTs for these stimuli. On the one hand, a regression analysis was carried out on pseudowords’ mean RTs of each participant across the six task blocks, separately for both training conditions. Results revealed significant linear trends for the mean RTs obtained by participants across blocks under both conditions (**Figure 1A**), although stronger in the semantic-associative training [*R*^2^ = 0.247, *F*(1,131) = 42.81, *p* < 0.001] than in the non-associative training context [*R*^2^ = 0.052, *F*(1,131) = 7.15 *p* < 0.01]. Thus, the mean RTs exhibited by pseudowords were gradually reduced as a consequence of repetitions, although this decrease in RTs was better explained by repetitions when pseudowords were trained under the semantic-associative context. On the other hand, a regression analysis was conducted on differences between pseudowords’ mean RTs obtained at both associative and non-associative contexts by each participant across the six task blocks. This analysis revealed a significant linear trend [*R*^2^ = 0.138, *F*(1,131) = 20.72, *p* < 0.001], showing that the gradual increase of differences between RTs exhibited by pseudowords at both conditions was explained by the variable repetition. The more the pseudowords were repeated across blocks, the higher were the differences between RTs obtained at both training conditions, due to meaningful associations carried out at the semantic-associative context (**Figure 1B**).

### Regression-Based ERPs (rERPs)

**FIGURE 2:**
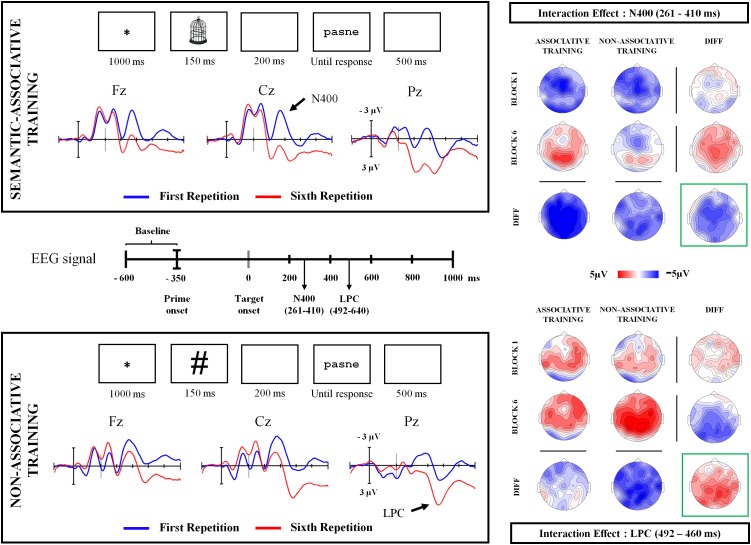
Sequence of stimuli presentation at both semantic-associative and non-associative training conditions. The latency of elements presented across the sequence is indicated by the numbers displayed at the bottom of each rectangle. Grand averaged ERP waveforms show a different modulation for both semantically associated and non-associated pseudowords across repetition blocks (black bold arrows). At the center of the figure, the time (in milliseconds) is represented, corresponding to the EEG signal recording. Arrows indicate the time when the onset of N400 and LPC components were found significant. Topographic maps for each interactive time window found significant in the analysis of the regression-based ERPs are plotted, showing the scalp distribution of the ERP activity for each condition as well as the differences between conditions (maps under or to the right of the label DIFF). Maps framed in green reflect the scalp distribution of the interactive effect at each time window.

**FIGURE 3:**
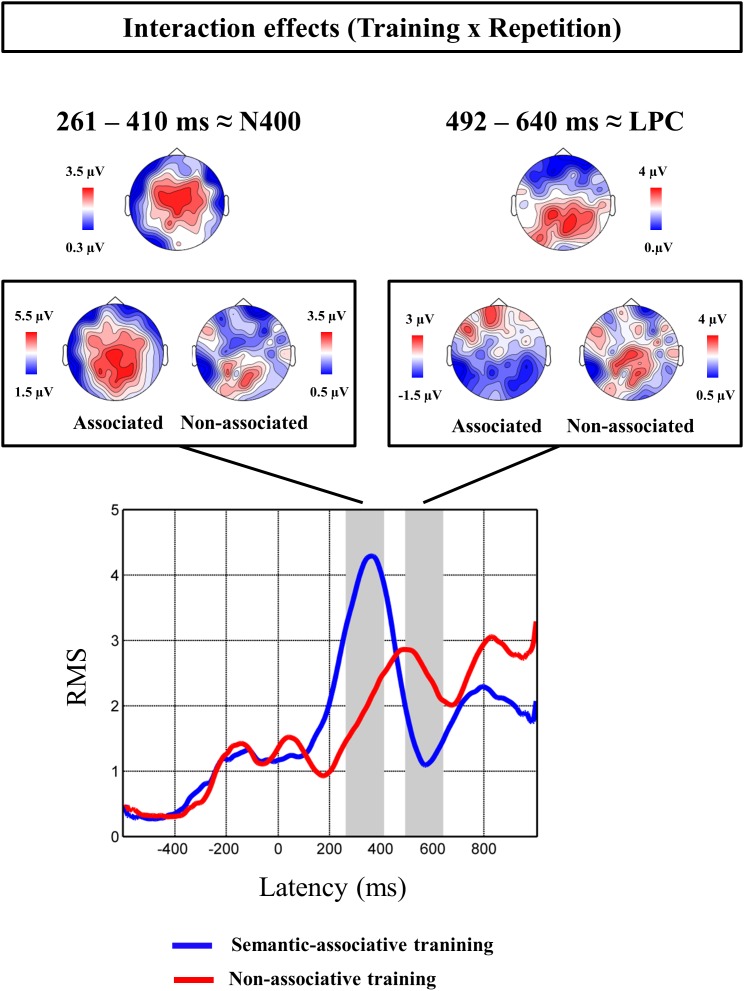
Interaction effects found significant at the regression-based ERP analysis. Topographic maps at the top of the figure show the scalp distribution of the interactive effects found at both N400 and LPC time windows. Both interactive effects are subsequently decomposed, showing the effect of repetition on both semantic-associative and non-associative conditions. Topographical maps framed show the distribution of the maximal activation for the repetition effect on each condition and temporal window. Root mean square (RMS) waveforms of regression-based ERPs are shown for pseudowords repeated under each training condition.

Grand averaged ERP waveforms indicated two amplitude changes across repetitions (**Figure 2**). First, there seem to be reductions in N400 amplitude for pseudowords presented under the semantic-associative training condition, with apparently smaller decreases for pseudowords non-associated to any stimulus. In contrast, increased LPC amplitude was observed only for pseudowords presented under the non-associative condition. The visual inspection of the root mean square (RMS) for interaction-related rERPs suggests that repetition and type of training interacted in time windows coinciding with N400 and LPC components (**Figure 3**).

Cluster analysis for interaction-related rERPs revealed different repetition effects for pseudowords under both conditions. Particularly, two significant clusters of effects were identified: one extended from 261 to 410 ms and showing a fronto-central distribution (*p* < 0.05), and another from 492 to 640 ms, and with a left temporo-parietal topography (*p* < 0.05). The latency and scalp distribution of the two clusters suggests that the interaction between repetition and type of training modulated instances of both the N400 and LPC components.

For the earlier N400-like cluster, the effect of repetition was found to be significant for pseudowords under both semantic-associative [*t*(21) = 4.5, *p* < 0.001] and non-associative conditions [*t*(21) = 3.33, *p* < 0.005], but greater for semantically associated (mean of 4.57 μV) than for non-associated pseudowords [mean of 1.56 μV; *t*(21) = 2.91, *p* < 0.01]. Thus, pseudoword repetition increased positive amplitudes globally, but with stronger increases for those pseudowords associated to pictures. In contrast, for the latter, LPC-like cluster, only pseudowords non-associated to any stimulus showed a significant effect of repetition, with more positive amplitudes after repetition [*t*(21) = 3.27, *p* < 0.005]. In addition, the repetition effect for isolated pseudowords (2.84 μV) differed significantly from that for semantically associated pseudowords [−0.32 μV; *t*(21) = −4.05, *p* < 0.005].

**FIGURE 4:**
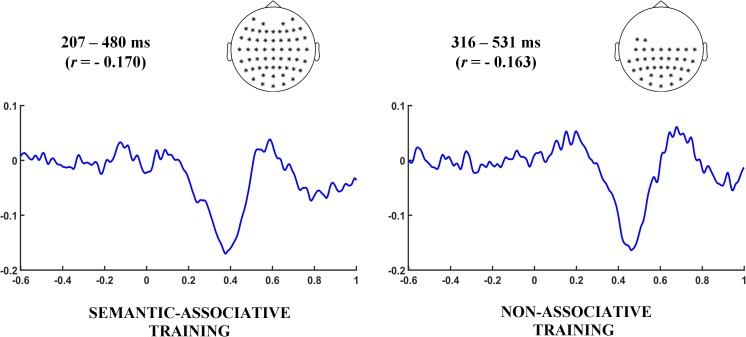
Time intervals and electrodes indicate the latency and spatial distribution in which correlation between behavioral and electrophysiological changes exhibited by pseudowords was found significant under each training condition. Correlation values are indicated in parentheses. The grand averaged correlation-based ERP waveforms were calculated taking the time interval and electrodes found significant under each training condition. A negative correlation around 400 ms after pseudoword onset can be observed at both conditions, slightly earlier for the semantic-associative training condition.

Subsequent single-trial correlation analyses were carried out to explore the relation between changes in electrophysiological and behavioral data across repetitions. First, EEG activity at every sample point (latency and electrode) was correlated for each participant to RTs using Spearman’s rho correlation coefficient. Correlations were calculated separately for pseudowords under both semantic-associative and non-associative conditions, using segments from −600 to 1000 ms after target onset. Next, the resulting correlation-based ERPs were contrasted against zero over the whole set of sample points by means of cluster-based permutation analysis. A cluster from 207 to 480 ms (*p* < 0.001) was found significant for semantically associated pseudowords whereas the analysis for non-associated pseudowords revealed a cluster in a later time window, from 316 to 531 ms (*p* < 0.001). Both effects indicate negative correlations between ERPs and RTs: as RTs reduce through repetitions, the electrophysiological activity becomes positive (maximum correlation values obtained for pseudowords under both semantic-associative and non-associative conditions were −0.170 and −0.163, respectively). These results suggest that the activity modulation better related to changes in the speed for categorizing both types of pseudowords occurred in temporal windows likely corresponding to N400, although in a slightly different latency. Nonetheless, it is not possible to ensure that the positivity enhancement that is better related to the decrease in RTs corresponds to the modulation of this particular component. Instead of that, this result only indicates that as the time for categorization responses decrease, the electrophysiological activity becomes positive. See **Figure 4** for correlation results under both semantic-associative and non-associative conditions.

### Traditional ERPs

The averaged activity at the corresponding time windows and electrodes where interactive clusters reached significance, namely, at 261–410 and 492–640 ms, was used to carry out traditional ERPs analysis. The aim of this procedure was to compare results obtained under both standard and regression-based ERP analysis. Repeated measures ANOVAs with type of training (associated vs. non-associated pseudowords) and repetition (first vs. sixth block) as within-subject factors found significant interactions at both N400 [*F*(1,21) = 5.69, *p* < 0.05, η^2^ = 0.21] and LPC time windows [*F*(1,21) = 16.10, *p* < 0.01, η^2^ = 0.43]. For the N400 time window, no differences between the two types of associated and non-associated pseudowords were found at the first block [*F*(1,21) = 0.026, *p* > 0.05, η^2^ = 0.01]. However, differences appeared at the sixth block of the task [*F*(1,21) = 7.9, *p* < 0.05, η^2^ = 0.27] as a consequence of the greater activity reduction for associated [*F*(1,21) = 18.59, *p* < 0.001, η^2^ = 0.47] than for non-associated pseudowords [*F*(1,21) = 12.38, *p* < 0.01, η^2^ = 0.37] after repetition. Regarding the LPC time window, the increase in the differences between the two types of stimuli from the first [*F*(1,21) = 1.79, *p* > 0.05, η^2^ = 0.07] to the sixth block of the task [*F*(1,21) = 5.20, *p* < 0.05, η^2^ = 0.19] was produced by the opposite effect. In this case, repetition caused an increase in positivity for non-associated pseudowords [*F*(1,21) = 12.23, *p* < 0.01, η^2^ = 0.36], whereas the activity related to semantically associated pseudowords was not affected by repetition [*F*(1,21) = 0.056, *p* > 0.05, η^2^ = 0.003].

Therefore, the pattern of results found for both N400-like and LPC-like components was confirmed by traditional ERP analyses. Furthermore, these complementary analyses verified that the effects detected by taking the repetition as a continuous variable, in the context of regression analyses, can be also found considering this variable as categorical.

## Discussion

In the present regression-based ERP study, the repetition effect of new word-like stimuli – pseudowords – was tested under a semantic-associative training context, in which pseudowords were preceded by a picture referring to a known concept, and under a non-associative training context, in which pseudowords were preceded by a hash mark. The aim was to explore the impact of these two different trainings on the online, gradual lexicalization of pseudowords. For this purpose, we focused on two ERP components whose modulation could indicate the episodic or lexico-semantic nature of the processing developed through both trainings, and therefore clarify whether the lexicalization process is possible after the simple visual repetition or, to the contrary, an association to a meaningful stimulus is required. Overall, we found that, reflected in the reduction of the N400 amplitude, the repeated semantic-associative exposure between pictures and pseudowords facilitated the lexico-semantic processing of these stimuli. In contrast, the simple visual exposure of pseudowords under a non-associative context increased LPC amplitudes, reflecting an improvement in their episodic rather than in their lexico-semantic processing. In what follows, ERP and behavioral findings for each training condition are discussed.

On the one hand, pseudoword repetition in the semantic-associative condition caused an amplitude reduction in an N400-like component, consistent with other studies reporting N400 reductions after repeated association of pseudowords with pictures or definitions or after embedding them in sentences with a constrained semantic context (Mestres-Missé et al., 2007; Dobel et al., 2009; Borovsky et al., 2010; Frishkoff et al., 2010; Batterink and Neville, 2011). In line with conclusions stated in these prior studies, this N400 modulation seems to indicate the facilitation in the lexico-semantic processing of the stimuli trained under the associative context. However, the possibility that this effect reflects also the involvement of other processes must be considered, taking into account the recent debate about the relation between N400 and semantic processes and, importantly, the similar N400 reduction that was also observed following the repetition of pseudowords under the non-associative condition, with no semantic information involved. In this regard, different recognition memory studies have found that visual repetition of stimuli produces the modulation of a frontal N400. Although there are some evidences against (Paller et al., 2007; Voss and Federmeier, 2011; Bermúdez-Margaretto et al., 2015), this so-called FN400 component has been considered functionally different from the typical centro-posterior N400 and stated as a marker of stimuli familiarity instead of related to semantic processes (Van Strien et al., 2005; Groh-Bordin et al., 2006; Laszlo and Federmeier, 2007; Rugg and Curran, 2007; Bridger et al., 2012; Laszlo et al., 2012). Accordingly, the N400 reduction found in the present study at both training contexts, which shows a fronto-central scalp distribution, could be reflecting the increase in the familiarity of the stimuli as a consequence of their visual experience across repetitions, rather than the facilitation in their lexico-semantic processing. Nonetheless, this interpretation must be considered carefully. In this sense, such modulation seems to be dependent not only on visual repetition but also on semantic information, given that the N400 reduction was significantly higher for those pseudowords repeated under the semantic-associative training. The higher N400 reduction found for these stimuli could reflect just the achievement of a greater familiarity as a consequence of repetitions under the associative context. However, it cannot be discarded that this N400 effect shows the enhancement in both the familiarity and the semantic facilitation of the stimuli as a result of the meaningful associations through repetitions.

Therefore, the present N400-like modulation found under the semantic-associative context could be interpreted in terms of a meaningful association between the concept given by the picture and the pseudoword throughout repetitions. This association could enhance both reading and posterior categorization of these stimuli, a response that could be predicted more quickly from the previous presentation of the picture. Behavioral data support this idea, given that reductions in RTs were found to be greater for pseudowords repeatedly associated to same pictures than for those pseudowords repeated under the non-associative condition, where presentation of these stimuli was not predictable from the previous mark. Indeed, differences in RTs obtained for pseudowords under both training conditions increased gradually through blocks and were found as early as in the first block, result that shows the extremely fast association reached between pictures and pseudowords in the semantic-associative context. The acquisition of this fast association could be explained by task requirements in the present study, namely, the lexical decisions. In this sense, systematic associations allowed participants to establish a prediction for the incoming stimulus and therefore increase the speed in their responses across blocks. Furthermore, the higher N400 modulation found in the associative training context likely shows the contribution of this association to the better integration of pseudowords into the picture-context given, facilitating their processing and speeding up their responses. This statement is supported by the negative correlation found between both behavioral and electrophysiological indexes, since as the activity became positive in a time window corresponding to N400, response times for pseudowords decreased.

Taking into account that pseudoword predictability increased across the associative training, the current N400 effect might instead be explained by differences in stimuli predictability between the two training contexts. This would be in line with more recent considerations about this component (Kutas et al., 2006; Kutas and Federmeier, 2011) and the key role of prediction in language comprehension (Coulson and Federmeier, 2002; DeLong et al., 2005; Federmeier, 2007). Specifically, N400 modulations are considered to reflect not only the enhancement of semantic relatedness between stimuli, but also the facilitation in their interpretation by means of contextual information. Thus, the more predictable the stimulus, the easier its interpretation results, with higher reductions in the N400 amplitude observable (Kutas and Van Petten, 1994; Dambacher et al., 2006; Federmeier et al., 2007; Lau et al., 2013). In the present study, the encounter of stimuli under the semantic-associative condition (pseudowords but also filler words) could be unequivocally predicted from the previous picture, with the predictive value for these stimuli increasing across expositions. Thus, semantic-cues facilitated the prediction of pseudowords across the task, resulting in higher N400 reductions. By contrast, the lack of correspondence between visual cues and pseudowords in the non-associative training condition was reflected by smaller N400 reductions. Importantly, although the encounter with pictures could be enough to carry out lexical decisions through the task, these results and also those obtained from correlation analyses show that pseudoword facilitation occurred *during* the time course of the target processing.

On the other hand, pseudoword repetition in the non-associative condition caused an increase in the LPC-like activity, which was not found under the semantic-associative condition. As other authors (Swick and Knight, 1996; Van Petten et al., 2000; Van Strien et al., 2005; Bermúdez-Margaretto et al., 2015) have already pointed out after repeated visual exposition, this LPC enhancement could be related to the construction and strengthening of episodic memory traces, facilitating the reading and categorization of the repeated stimuli. In this sense, the enhanced processing of pseudowords under the non-associative condition was also reflected by progressive reductions in RTs observed across repetitions. The presentation of these pseudowords was not preceded by a specific stimulus; hence, their presentation could not unequivocally be predicted by the previous mark. This resulted in greater monitoring and slower processing of these stimuli in comparison to those associated to pictures. Despite this fact, repeated visual exposure to these stimuli facilitated their processing, as indicated by both behavioral and electrophysiological data.

Thus, the modulation of the LPC-like activity through the simple visual repetition of pseudowords would indicate the achievement of an episodic processing for the stimuli under the non-associative training condition, which facilitates their categorization across the task. Interestingly, this LPC modulation was not found through pseudoword repetition under the semantic-associative training condition. This likely implies that the processing of these stimuli relied more upon predictions than on episodic retrieval. Indeed, the increase of predictability for these stimuli as a consequence of their repeated association with meaningful cues caused a significant drop in RTs, which decreased to ∼400 ms on average. This shift in RTs could induce a shift in ERP latency, masking thereby the LPC effect in this semantic-associative condition. Other studies (Perfetti et al., 2005; Batterink and Neville, 2011; Bakker et al., 2015) have reported enhanced LPC activity following repetition of pseudowords embedded in semantic-associative contexts. The difference with the present study could be due to the amount of semantic demand involved in the respective tasks. In this sense, LPC enhancements are usually found in tasks where, instead of lexical decisions, an explicit semantic judgment about trained stimuli is required. Therefore, even though LPC modulation by repetition might be expected regardless of the sort of training, the association between pictures and pseudowords, along with the lack of an explicit semantic processing, seems to reduce the burden at retrieval and recognition processes thought to underlie LPC activity – and likely causes the masking of the effect.

To the best of our knowledge, this is the first electrophysiological study that directly compares the different brain activity modulation produced by repeated exposure of new (pseudo-) words under semantic-associative and non-associative training contexts, providing information about the contribution of each of these trainings in processes that take part in the lexicalization of the new stimuli. While some authors have claimed that a rich and deep training involving not only visual repetition but also meaningful information about new words is required for achievement of an interactive processing that allows their complete lexicalization (Leach and Samuel, 2007; Qiao and Forster, 2013; Qiao et al., 2013), others have argued that the simple visual exposure to new words is sufficient to acquire a lexical processing for these stimuli (Maloney et al., 2009; Kwok and Ellis, 2015; Suárez-Coalla et al., 2016; Kwok et al., 2016). The results reported in the present study could support the claim that repeated visual exposure to novel words in association with meaningful stimuli constitutes a more advantageous strategy for their lexicalization than simple visual exposure. Nonetheless, the interpretation of the N400 modulation obtained in the semantic-associative condition must be approached with caution. This effect likely indicates the increase in both the familiarity and the semantic facilitation of the stimuli as a consequence of the acquisition of a processing guided and facilitated by predictability reached through the repeated semantic-association between consistent meaningful-cues and new orthographic labels across the task. However, not only the on-line tracing of effects produced under both trainings, but also a post-training test would be required in a future study to ensure the existence and nature of novel word representations, collecting both behavioral and electrophysiological measures which evaluate the interactive processing of trained words into the linguistic system of readers.

## Conclusion

The multiple linear regression analyses applied here confirm the usefulness of this approach in disentangling simultaneous effects of different but inter-correlated variables in brain response, in this case the semantic and visual training of new word-like stimuli under semantic-associative and non-associative contexts. Overall, a general advantage is found in the processing of pseudowords under the semantic-associative training context, as could be observed in the larger decrease in RTs and in the N400 amplitude throughout the task. However, the explicit categorization responses carried out by participants could influence the training and cause uncertain effects not related to reading, but rather to motor or decisional processes. To clarify this question, new studies are needed in which the effects of the semantic-associative and non-associative training of pseudowords are tested in tasks not involving decision-making about the stimuli.

## Author Contributions

Use of ERPs for studying the neurobiology of reading processing and, particularly, the electrophysiological signatures of the novel word learning. Implementation of different techniques for EEG signal analysis, as cluster-based non-parametric techniques, multiple regression analysis of ERPs, or neural source localization, using softwares as Analyzer, Cartool, or Matlab. Participation in different research projects on which the main topics were the cognitive, neuropsychological, and electrophysiological changes during novel word lexicalization.

## Conflict of Interest Statement

The authors declare that the research was conducted in the absence of any commercial or financial relationships that could be construed as a potential conflict of interest.

## References

[B1] Angwin A. J. Phua B. Copland D. A. (2014). Using semantics to enhance new word learning: an ERP investigation. *Neuropsychologia* 59 169–178. 10.1016/j.neuropsychologia.2014.05.002: 24846835

[B2] Bakker I. Takashima A. van Hell J. G. Janzen G. McQueen J. M. (2015). Tracking lexical consolidation with ERPs: lexical and semantic-priming effects on N400 and LPC responses to newly-learned words. *Neuropsychologia* 79 33–41. 10.1016/j.neuropsychologia.2015.10.020 26476370

[B3] Batterink L. Neville H. (2011). Implicit and explicit mechanisms of word learning in a narrative context: an event-related potential study. *J. Cogn. Neurosci.* 23 3181–3196. 10.1162/jocn_a_00013 21452941 PMC3129368

[B4] Bentin S. (1987). Event-related potentials, semantic processes, and expectancy factors in word recognition. *Brain Lang.* 31 308–327. 10.1016/0093-934X(87)90077-0 3620905

[B5] Bermúdez-Margaretto B. Beltrán D. Dominguez A. Cuetos F. (2015). Repeated exposure to “meaningless” pseudowords modulates LPC, but not N (FN) 400. *Brain Topogr.* 28 838–851. 10.1007/s10548-014-0403-5 25266047

[B6] Borovsky A. Kutas M. Elman J. (2010). Learning to use words: event-related potentials index single-shot contextual word learning. *Cognition* 116 289–296. 10.1016/j.cognition.2010.05.004 20621846 PMC2904319

[B7] Bowers J. S. Davis C. J. Hanley D. A. (2005). Interfering neighbours: the impact of novel word learning on the identification of visually similar words. *Cognition* 97 B45–B54. 10.1016/j.cognition.2005.02.002 15925358

[B8] Breitenstein C. Zwitserlood P. de Vries M. H. Feldhues C. Knecht S. Dobel C. (2007). Five days versus a lifetime: intense associative vocabulary training generates lexically integrated words. *Restor. Neurol. Neurosci. 25* 5 493–500. 18334767

[B9] Bridger E. K. Bader R. Kriukova O. Unger K. Mecklinger A. (2012). The FN400 is functionally distinct from the N400. *Neuroimage* 63 1334–1342. 10.1016/j.neuroimage.2012.07.047 22850570

[B10] Clay F. Bowers J. S. Davis C. J. Hanley D. A. (2007). Teaching adults new words: the role of practice and consolidation. *J. Exp. Psychol.* 33:970. 10.1037/0278-7393.33.5.970 17723073

[B11] Coltheart M. Rastle K. Perry C. Langdon R. Ziegler J. (2001). DRC: a dual route cascaded model of visual word recognition and reading aloud. *Psychol. Rev.* 108 204–256. 10.1037/0033-295X.108.1.204 11212628

[B12] Coulson S. Federmeier K. D. (2002). *Words in Context: ERPs and the Lexical/Postlexical Distinction*. Available at: http://www.cogsci.ucsd.edu/coulson/jpr.htm

[B13] Dambacher M. Kliegl R. Hofmann M. Jacobs A. M. (2006). Frequency and predictability effects on event-related potentials during reading. *Brain Res.* 1084 89–103. 10.1016/j.brainres.2006.02.010 16545344

[B14] Davis C. Perea M. (2005). BuscaPalabras: a program for deriving orthographic and phonological neighborhood statistics and other psycholinguistic indices in Spanish. *Behav. Res. Methods* 37 665–671. 10.3758/BF03192738 16629300

[B15] DeLong K. A. Urbach T. P. Kutas M. (2005). Probabilistic word pre-activation during language comprehension inferred from electrical brain activity. *Nat. Neurosci.* 8 1117–1121. 10.1038/nn1504 16007080

[B16] Dobel C. Junghöfer M. Breitenstein C. Klauke B. Knecht S. Pantev C. (2009). New names for known things: on the association of novel word forms with existing semantic information. *J. Cogn. Neurosci.* 22 1251–1261. 10.1162/jocn.2009.21297 19583468

[B17] Dumay N. Gaskell M. G. (2007). Sleep-associated changes in the mental representation of spoken words. *Psychol. Sci.* 18 35–39. 10.1111/j.1467-9280.2007.01845.x 17362375

[B18] Ellis A. W. Ferreira R. Cathles-Hagan P. Holt K. Jarvis L. Barca L. (2009). Word learning and the cerebral hemispheres: from serial to parallel processing of written words. *Philos. Trans. R. Soc. Lon. B Biol. Sci.* 364 3675–3696. 10.1098/rstb.2009.0187 19933140 PMC2846318

[B19] Federmeier K. D. (2007). Thinking ahead: the role and roots of prediction in language comprehension. *Psychophysiology* 44 491–505. 10.1111/j.1469-8986.2007.00531.x 17521377 PMC2712632

[B20] Federmeier K. D. Wlotko E. W. De Ochoa-Dewald E. Kutas M. (2007). Multiple effects of sentential constraint on word processing. *Brain Res.* 1146 75–84. 10.1016/j.brainres.2006.06.101 16901469 PMC2704150

[B21] Friedman D. (1990). Cognitive event-related potential components during continuous recognition memory for pictures. *Psychophysiology* 27 136–148. 10.1111/j.1469-8986.1990.tb00365.x 2247545

[B22] Frishkoff G. A. Perfetti C. A. Collins-Thompson K. (2010). Lexical quality in the brain: ERP evidence for robust word learning from context. *Dev. Neuropsychol.* 35 376–403. 10.1080/87565641.2010.480915 20614356 PMC2906764

[B23] Groh-Bordin C. Zimmer H. D. Ecker U. K. (2006). Has the butcher on the bus dyed his hair? When color changes modulate ERP correlates of familiarity and recollection. *Neuroimage* 32 1879–1890. 10.1016/j.neuroimage.2006.04.215 16777433

[B24] Hauk O. Davis M. H. Ford M. Pulvermüller F. Marslen-Wilson W. D. (2006). The time course of visual word recognition as revealed by linear regression analysis of ERP data. *Neuroimage* 30 1383–1400. 10.1016/j.neuroimage.2005.11.048 16460964

[B25] Hauk O. Pulvermüller F. Ford M. Marslen-Wilson W. D. Davis M. H. (2009). Can I have a quick word? Early electrophysiological manifestations of psycholinguistic processes revealed by event-related regression analysis of the EEG. *Biol. Psychol.* 80 64–74. 10.1016/j.biopsycho.2008.04.015 18565639

[B26] Kutas M. Federmeier K. D. (2011). Thirty years and counting: finding meaning in the N400 component of the event related brain potential (ERP). *Annu. Rev. Psychol.* 62 621–647. 10.1146/annurev.psych.093008.131123 20809790 PMC4052444

[B27] Kutas M. Hillyard S. A. (1984). Brain potentials during reading reflect word expectancy and semantic association. *Nature* 307 161–163. 10.1038/307161a0 6690995

[B28] Kutas M. Van Petten C. K. (1994). “Psycholinguistics electrified: event-related brain potential investigations,” in *Handbook of Psycholinguistics*, ed. Gernsbacher M. A. (San Diego, CA: Academic Press), 83–143.

[B29] Kutas M. Van Petten C. K. Kluender R. (2006). “Psycholinguistics electrified II (1994–2005),” in *Handbook of Psycholinguistics*, 2nd Edn, eds Traxler M. J. Gernsbacher M. A. (New York, NY: Elsevier), 659–724.

[B30] Kwok R. K. W. Cuetos F. Avdyli R. Ellis A. W. (2016). Reading and lexicalization in opaque and transparent orthographies: word naming and word learning in English and Spanish. *Quart. J. Exp. Psychol.* 70 2105–2129. 10.1080/17470218.2016.1223705 27609455

[B31] Kwok R. K. W. Ellis A. W. (2015). Visual word learning in skilled readers of English. *Quart. J. Exp. Psychol.* 68 326–349. 10.1080/17470218.2014.944549 25019273

[B32] Laszlo S. Federmeier K. D. (2007). Better the DVL you know: acronyms reveal the contribution of familiarity to single-word reading. *Psychol. Sci.* 18 122–126. 10.1111/j.1467-9280.2007.01859.x 17425530 PMC2692048

[B33] Laszlo S. Stites M. Federmeier K. D. (2012). Won’t get fooled again: an event-related potential study of task and repetition effects on the semantic processing of items without semantics. *Lang. Cognit. Process.* 27 257–274. 10.1080/01690965.2011.606667 22518068 PMC3328294

[B34] Lau E. F. Holcomb P. J. Kuperberg G. R. (2013). Dissociating N400 effects of prediction from association in single-word contexts. *J. Cogn. Neurosci.* 25 484–502. 10.1162/jocn_a_00328 23163410 PMC3657387

[B35] Leach L. Samuel A. G. (2007). Lexical configuration and lexical engagement: when adults learn new words. *Cognit. Psychol.* 55 306–353. 10.1016/j.cogpsych.2007.01.001 17367775 PMC2134982

[B36] Maloney E. Risko E. F. O’Malley S. Besner D. (2009). Tracking the transition from sublexical to lexical processing: on the creation of orthographic and phonological lexical representations. *Quart. J. Exp. Psychol.* 62 858–867. 10.1080/17470210802578385 19107643

[B37] Maris E. Oostenveld R. (2007). Nonparametric statistical testing of EEG-and MEG-data. *J. Neurosci. Methods* 164 177–190. 10.1016/j.jneumeth.2007.03.024 17517438

[B38] Mestres-Missé A. Rodriguez-Fornells A. Münte T. F. (2007). Watching the brain during meaning acquisition. *Cereb. Cortex* 17 1858–1866. 10.1093/cercor/bhl094 17056648

[B39] Oldfield R. C. (1971). The assessment and analysis of handedness: the edinburgh inventory. *Neuropsychologia* 9 97–113. 10.1016/0028-3932(71)90067-4 5146491

[B40] Oostenveld R. Fries P. Maris E. Schoffelen J.-M. (2011). FieldTrip: open source software for advanced analysis of MEG, EEG, and invasive electrophysiological data. *Comput. Intell. Neurosci.* 2011:156869. 10.1155/2011/156869 21253357 PMC3021840

[B41] Paller K. A. Voss J. L. Boehm S. G. (2007). Validating neural correlates of familiarity. *Trends Cognit. Sci.* 11 243–250. 10.1016/j.tics.2007.04.002 17475539

[B42] Perfetti C. A. Hart L. (2002). The lexical quality hypothesis. *Precursors Funct. Liter.* 11 67–86. 10.1075/swll.11.14per

[B43] Perfetti C. A. Wlotko E. W. Hart L. A. (2005). Word learning and individual differences in word learning reflected in event-related potentials. *J. Exp. Psychol. Learn. Mem. Cognit.* 31 1281–1292. 10.1037/0278-7393.31.6.1281 16393047

[B44] Qiao X. Forster K. Witzel N. (2013). Is banara really a word? *Cognition* 113 254–257. 10.1016/j.cognition.2009.08.006 19717145

[B45] Qiao X. Forster K. I. (2013). Novel word lexicalization and the prime lexicality effect. *J. Exp. Psychol. Learn. Mem. Cognit.* 39 1064–1074. 10.1037/a0030528 23088548

[B46] Rugg M. D. Curran T. (2007). Event-related potentials and recognition memory. *Trends Cognit. Sci.* 11 251–257. 10.1016/j.tics.2007.04.004 17481940

[B47] Schneider W. Eschman A. Zuccolotto A. (2002). *E-Prime (Version 2.0). [Computer Software and Manual]*. Pittsburgh, PA: Psychology Software Tools.

[B48] Share D. L. (1995). Phonological recoding and self-teaching: sine qua non of reading acquisition. *Cognition* 55 151–218. 10.1016/0010-0277(94)00645-2 7789090

[B49] Share D. L. (1999). Phonological recoding and orthographic learning: a direct test of the self-teaching hypothesis. *J. Exp. Child Psychol.* 72 95–129. 10.1006/jecp.1998.2481 9927525

[B50] Smith N. J. Kutas M. (2015). Regression-based estimation of ERP waveforms: I. The rERP framework. *Psychophysiology* 52 157–168. 10.1111/psyp.12317 25141770 PMC5308234

[B51] Snodgrass J. G. Vanderwart M. (1980). A standardized set of 260 pictures: norms for name agreement, image agreement, familiarity, and visual complexity. *J. Exp. Psychol. Hum. Learn. Mem.* 6 174–215. 10.1037/0278-7393.6.2.174 7373248

[B52] Suárez-Coalla P. Álvarez-Cañizo M. Cuetos F. (2016). Orthographic learning in Spanish children. *J. Res. Read.* 39 292–311. 10.1111/1467-9817.1204 25056668

[B53] Swick D. Knight R. T. (1996). Is prefrontal cortex involved in cued recall? A neuropsychological test of PET findings. *Neuropsychologia* 34 1019–1028. 10.1016/0028-3932(96)00011-5 8843069

[B54] Tamura N. Castles A. Nation K. (2017). Orthographic learning, fast and slow: lexical competition effects reveal the time course of word learning in developing readers. *Cognition* 163 93–102. 10.1016/j.cognition.2017.03.002 28314178

[B55] Van Petten C. (1993). A comparison of lexical and sentence-level context effects in event-related potentials. *Lang. Cognit. Process.* 8 485–531. 10.1080/01690969308407586

[B56] Van Petten C. Senkfor A. J. Newberg W. M. (2000). Memory for drawings in locations: spatial source memory and event-related potentials. *Psychophysiology* 37 551–564. 10.1017/S0048577200990541 10934914

[B57] Van Strien J. W. Hagenbeek R. E. Stam C. J. Rombouts S. A. R. B. Barkhof F. (2005). Changes in brain electrical activity during extended continuous word recognition. *NeuroImage* 26 952–959. 10.1016/j.neuroimage.2005.03.003 15955505

[B58] Voss J. L. Federmeier K. D. (2011). FN400 potentials are functionally identical to N400 potentials and reflect semantic processing during recognition testing. *Psychophysiology* 48 532–546. 10.1111/j.1469-8986.2010.01085.x 20701709 PMC2982896

[B59] Wang H. C. Savage G. Gaskell M. G. Paulin T. Robidoux S. Castles A. (2017). Bedding down new words: sleep promotes the emergence of lexical competition in visual word recognition. *Psychon. Bullet. Rev.* 24 1186–1193. 10.3758/s13423-016-1182-7 27785682

[B60] Weekes B. S. (1997). Differential effects of number of letters on word and nonword naming latency. *Quart. J. Exp. Psychol. A Hum. Exp. Psychol.* 50A, 439–456. 10.1080/027249897392170

